# The diagnostic accuracy of MRI in determining the relations between paraclinoid aneurysms and the cavernous sinus

**DOI:** 10.1007/s00234-021-02864-y

**Published:** 2021-11-25

**Authors:** Sérgio Tadeu Fernandes, Hugo Leonardo Doria-Netto, Raphael Vicente Alves, Renan Luiz Lapate, Nelson Paes Fortes Diniz Ferreira, Manoel Jacobsen Teixeira, Davi Jorge Fontoura Solla, Vitor Nagai Yamaki, Eberval Gadelha Figueiredo

**Affiliations:** 1 Divisão de Neurocirurgia Vascular, Hospital de Transplantes Do Estado de São Paulo Euryclides de Jesus Zerbini, Av. Brigadeiro Luiz Antônio 2651, São Paulo, SP Brazil; 2 Setor de Exames Diagnósticos, Hospital Do Coração, Paraíso, São Paulo, SP Brazil; 3 Alta Excelência Diagnóstica, Ibirapuera, São Paulo, SP Brazil; 4 Departamento de Neurologia, Divisão de Neurocirugia, Faculdade de Medicina, Universidade de São Paulo, São Paulo, SP Brazil

**Keywords:** Differential diagnosis, Internal carotid artery, Intertechnique agreement, Intracranial aneurysms, Subarachnoid hemorrhage

## Abstract

**Purpose:**

The location of paraclinoid aneurysms is determinant for evaluation of its intradural compartment and risk of SAH after rupture. Advanced MRI techniques have provided clear visualization of the distal dural ring (DDR) to determine whether an aneurysm is intracavernous, transitional or intradural for decision-making. We analyzed the diagnostic accuracy of MRI in predicting whether a paraclinoid aneurysm is intracavernous, transitional or intradural.

**Methods:**

We conducted a prospective cohort between January 2014 and December 2018. Patients with paraclinoid aneurysms underwent 3D fast spin-echo MRI sequence before surgical treatment. The DDR was the landmark for MRI characterization of the aneurysms as follow: (i) Intradural; (ii) Transitional; and (iii) Intracavernous. The MRI sensitivity, specificity, positive and negative likelihood ratios were determined compared to the intraoperative findings. We also evaluated the intertechnique agreement using the Cohen’s kappa coefficient (κ) for dichotomous classifications (cavernous vs non-cavernous).

**Results:**

Twenty patients were included in the cohort. The accuracy of MRI showed a sensitivity of 86.7% (95%CI:59.5–98.3) and specificity of 90.0% (95%CI:55.5–99.8). Analyzing only patients without history of SAH, accuracy test improved with a sensitivity of 92.3% (95%CI:63.9–99.8) and specificity reached 100% (95%CI: 63–100). Values of Cohen’s kappa (κ), intertechnique agreement was considered substantial for dichotomous classifications (κ = 0.754; *p* < 0.001). For patients without previous SAH, intertechnique agreement was even more coincident for the dichotomous classification (κ = 0.901; *p* < 0.001).

**Conclusion:**

3D fast spin-echo MRI sequence is a reliable and useful technique for determining the location of paraclinoid aneurysms in relation to the cavernous sinus, particularly for patients with no history of SAH.

## Introduction

Cerebral aneurysms located in the intradural compartment are at higher risk of rupture and often require endovascular/microsurgical treatment. In contrast, aneurysms located within the cavernous sinus rarely manifest with SAH and have lower morbidity and mortality. Unfortunately, brain-imaging techniques generally do not disclose whether the clinoid segment (C5) is intracavernous or non-cavernous [1–12]. Hence, it is not always possible to achieve an accurate topographic diagnosis of brain aneurysms located in this segment of the ICA regarding their relationship with the carotid collar, the proximal dural ring (PDR) and the distal dural ring (DDR), or to determine if the aneurysm is intracavernous, transitional or intradural [9, 13–18].

Several studies have suggested that complementary MRI is effective in predicting the location of paraclinoid aneurysms [11, 13, 15, 16, 18, 19]. However, most studies have not provided sufficient evidence regarding the agreement between MRI and intraoperative findings. On this basis, we analyzed the diagnostic accuracy of MRI in predicting whether a paraclinoid aneurysm is intracavernous, transitional or intradural.

## Material and methods

### Study design

This is a cohort comprising individuals recruited between January/2014 and December/2018. The study was approved by the Institutional Review Board (protocol no. 456.498–10/03/2103). Informed consent form was assigned for all participants.

### Study population

The minimum sample size required to achieve the 95% confidence level (95%CI) was determined to be 16 according to the following equation:

$$\mathrm{Sample}\;\mathrm{size}\;(\mathrm n)=\lbrack\mathrm{DEFF}\ast\mathrm{Np}(1-\mathrm p)\rbrack/\lbrack(\mathrm d2/\mathrm Z21-\alpha/2\ast(\mathrm N-1)+ p\ast(1-\mathrm p)\rbrack$$

DEFF: design effect (taken as 1.0 for random sampling)
N: sample size
p: assumed hypothetical frequency of aneurysms in the population (1%)
d: desired absolute precision (0.05)
Z: critical value for 95%CI.

The inclusion criteria were: (i) the presence of multiple aneurysms with at least one classified as paraclinoid; or (ii) the presence of a single paraclinoid aneurysm. The exclusion criteria were: (i) MRI contraindications; (ii) contraindications for microsurgical treatment; (iii) presence of a single aneurysm unambiguously classified as intracavernous without previous SAH; and (iv) pregnancy. Of note: (i) the primary objective of all procedures was the decision to treat and planning of microsurgical clipping of paraclinoid aneurysms; (ii) the authors understand the current practice preferentially recommends the endovascular approach for paraclinoid aneurysms. However, the Brazilian public health system does not routinely afford flow diverter devices, providing a window of opportunity to investigate this issue, which is relevant regardless of the treatment method.

### Acquisition of MRI data

Coronal T2-weighted images were acquired on a MAGNETOM® Verio 3 Tesla (3 T) MRI scanner (Siemens, Erlangen/Germany) with an 8-channel head coil using a 3D fast spin-echo sequence. The spatial matrix was obtained using the sensitivity encoding technique with the parameters set as follows: repetition time, 4700–6550 ms; echo time, 104.6–110.6 ms; field-of-view, 18–20 × 18–20 cm; slice thickness, 2 mm, space/gap, 0 mm; matrix size, 448 × 448, number of excitations, 4; echo train length, 17.

Images were analyzed by a single neuroradiologist with more than 20 years of experience in order to identify the PDR, the DDR and adjacent anatomical landmarks, the paraclinoid aneurysm and the relationship of the neck and dome of the aneurysm with adjacent structures. The DDR was the landmark for MRI characterization. It was identified as the reflection of the dura mater that surrounds the ICA at the point of exit from the cavernous sinus roof [3, 4, 16–18].

The positions of paraclinoid aneurysms in relation to the DDR were classified as follows: (i) intradural — both neck and dome located distally to the DDR; (ii) transitional — neck or dome (but not both) located distally to the DDR; and (iii) intracavernous (extradural) — both neck and dome located proximally to the DDR [9, 14, 16, 18, 20]. Aneurysms were further classified based on the spatial orientation of the dome in relation to the roof of the cavernous sinus as superior, medial, lateral or inferior as previously described by Krisht and Hsu [21].

### Microsurgical procedure

The patient was placed in a horizontal supine position with the head turned contralaterally for a pterional craniotomy. Microscopic dissection exposed the sphenoidal segment of the middle cerebral artery, the proximal segment of the anterior cerebral artery and the entire ICA supraclinoid segment with all the associated cisterns and nerves in order to allow visualization of the dura mater covering the anterior clinoid process (ACP).

Anterior clinoidectomy extended from the medial border of the optic nerve to the lateral margin of the lesser sphenoid wing over the superior orbital fissure and 1 cm from the posterior margin of the optic canal. The ACP tip was disconnected from the sphenoid and detached from the carotid-oculomotor membrane. Osteotomy of the optic strut was performed when needed and fibrin glue injection was used as necessary to reduce bleeding from the cavernous sinus and allow clinoid triangle clear definition [22, 23].

Patients presenting aneurysms unambiguously intradural or transitional, dissection through the neck proceeded for aneurysms clipping. The surgical procedure was recorded for analysis.

### Statistical analysis

Categorical variables were described as absolute and relative frequencies and compared using the χ2 test. Continuous variables were assessed for normality using graphical methods and skewness/kurtosis statistics. Age was expressed as mean ± standard deviation and compared using independent samples t-test. Aneurysm sizes were expressed as median and quartiles and compared using the Mann–Whitney test. In order to evaluate the accuracy of MRI screening with respect to the location of paraclinoid aneurysms, taking intraoperative findings as gold standard, a dichotomous classification was adopted. Transitional and intradural locations were merged into the non-cavernous group. Sensitivity, specificity, positive and negative likelihood ratios (PLR and NLR, respectively), and positive and negative predictive values (PPV and NPV) were calculated. The agreement between the results of MRI screening and intraoperative findings was evaluated for both trichotomous (i.e. intracavernous, transitional and intradural) and dichotomous classifications according to Cohen’s kappa coefficient (κ) with the following interpretations: 0 (no agreement); 0.10–0.19 (poor); 0.20–0.39 (fair); 0.40–0.59 (moderate); 0.60–0.79 (substantial); 0.80–0.99 (almost perfect) and 1.00 (perfect). Statistical significance was set at 0.05 and the tests were two-tailed. All analyses were performed with the aid of IBM SPSS software version 24.0 (Armonk/NY/USA) and MedCalc Statistical software version 15.2.0.0 (Ostend/Belgium).

## Results

### Characteristics of the sample population

Twenty-two patients were eligible and 20 (1 male and 19 females) were ultimately included. One did not undergo surgery within the study time frame and another was excluded due to MRI acquisition inadequacies.

The mean age was 51.4 ± 11.5 years. Fifteen (75%) participants presented ≥ 2 aneurysms with five patients having multiple paraclinoid aneurysms. A total of 25 paraclinoid aneurysms were considered individually in the analyses. Table 1 describes the sample.

**Table 1:** Characteristics of 20 patients of the cohort and of 25 paraclinoid aneurysms studied

Patients					Paraclinoid aneurysms			
Code	Age (years)	Gender	SAH history	Code	Location^a^		Size (mm)	Spatial orientation^b^
					MRI	M		
A	61	Female	No	#1	I	I	8	M
				#2	C	C	3	I
B	52	Female	Yes	#3	C	C	2	S
C	41	Female	No	#4	C	C	7	M
D	44	Female	Yes	#5	C	T	6	M
E	38	Female	Yes	#6	I	T	5	M
F	65	Female	No	#7	C	C	5	M
G	65	Female	No	#8	I	I	11	L
				#9	I	I	5	S
H	55	Female	No	#10	I	I	5	S
				#11	I	I	18	M
I	56	Female	No	#12	I	I	4	S
J	38	Female	Yes	#13	T	C	5	M
K	33	Female	No	#14	C	C	3	S
L	66	Female	No	#15	I	I	4	M
M	40	Female	No	#16	I	I	5	S
				#17	I	I	3	L
N	34	Female	No	#18	C	C	4	M
O	47	Female	No	#19	C	C	5	M
P	53	Male	No	#20	C	I	4	M
Q	49	Female	No	#21	I	I	3	M
				#22	I	I	4	S
R	73	Female	No	#23	C	C	6	M
S	63	Female	No	#24	I	I	5	M
T	41	Female	No	#25	C	C	5	M

^a^ Intracavernous (C), transitional (T) and intradural (I) locations determined by MRI or microsurgery (M)

^b^ Spatial orientations of the dome in relation to the cavernous sinus roof classified according to Krisht and Hsu as superior (S), medial (M), lateral (L) or inferior (I)

### Characteristics of paraclinoid aneurysms

The median aneurysm size was 5.0 mm (range 3–18 mm). Patients presenting with intracavernous or non-cavernous aneurysms had similar age (*p* = 0.313). The spatial orientation of the aneurysms in relation to the roof of the cavernous sinus were: 60% (15/25) medial projection, 28% (7/25) superior, 8% (2/25) lateral, and 4% (1/25) inferior (Tables 1 and 2).

**Table 2:** Characteristics of 20 patients of the cohort and size, location and spatial orientation of 25 paraclinoid aneurysms studied

Variable	Value	Location of aneurisms^a^		*P* value
		Intracavernous (*n* = 10)	Non-cavernous (*n* = 15)	
Age (years); mean ± standard deviation	51.4 ± 11.5	48.5 ± 13.8	53.3 ± 9.7	0.313^b^
Number of aneurysms in females (percentage of total)	24/25 (96)	10/10 (100)	14/15 (93.3)	1.000^c^
Size of aneurysms (mm); median (interquartile range)	5.0 (4.0–5.5)	5.0 (3.0–5.3)	5.0 (4.0–6.0)	0.511^d^
Spatial orientation^e^ of aneurysms; number (percentage)				0.322^c^
Superior	7/25 (28)	2/10 (20)	5/15 (33.3)	
Medial	15/25 (60)	7/10 (70)	8/15 (53.3)	
Lateral	2/25 (8)	0	2/15 (13.3)	
Inferior	1/25 (4)	1/10 (10)	0	

^a^ Non-cavernous aneurysms comprised the intradural and transitional types

^b^ Not significant according to Student's *t* test (*p* > 0.05)

^c^ Not significant according to χ^2^ test (*p* > 0.05)

^d^ Not significant according to Mann–Whitney test (*p* > 0.05)

^e^ Classified as described by Krisht and Hsu

### MRI screening vs. microsurgical findings

**Fig. 1:**
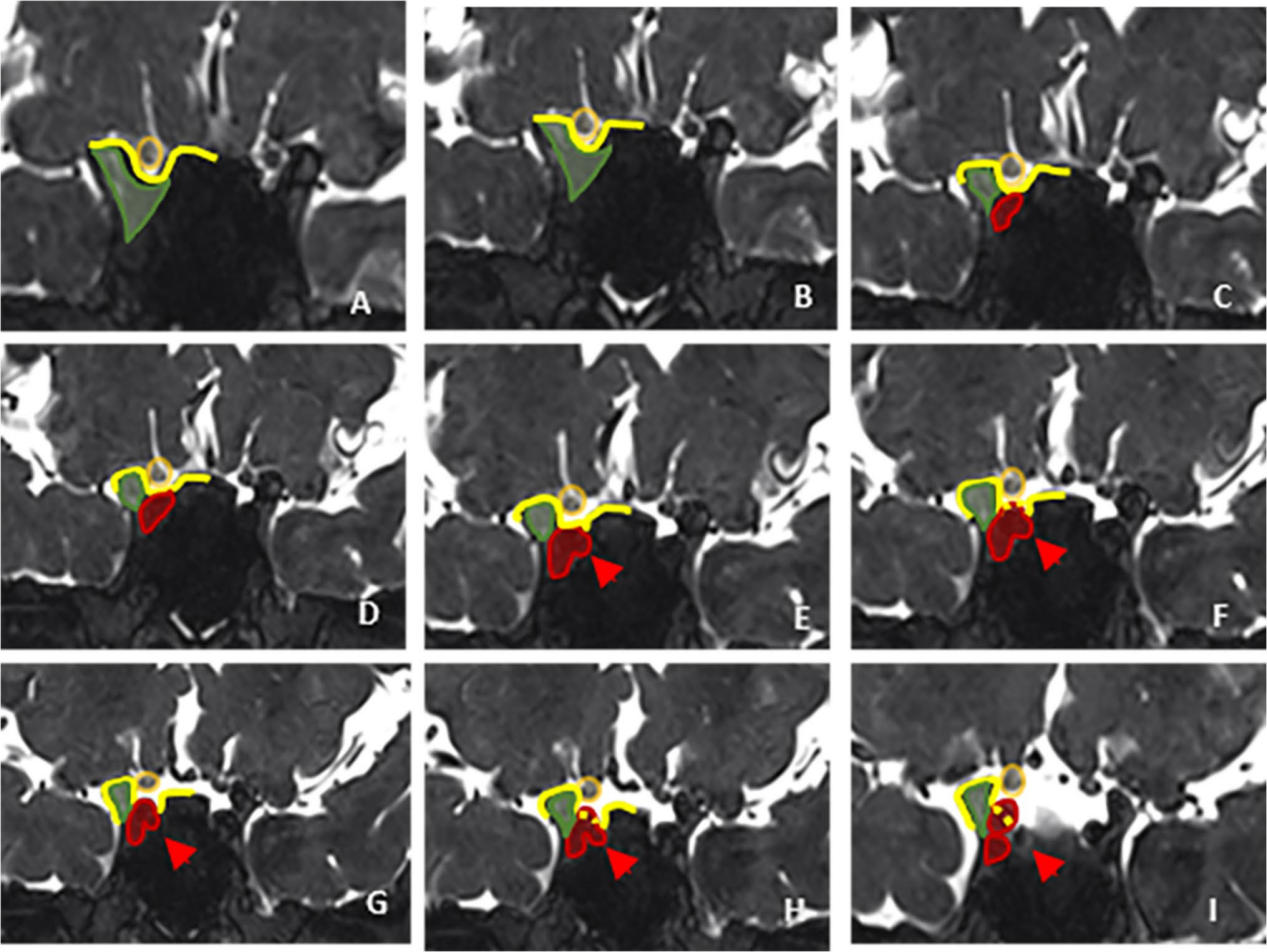
Coronal T2-weighted magnetic resonance imaging (MRI) scans acquired with a 3D fast spin-echo sequence showing anterior to posterior views of the anatomoradiological markers of the paraclinoid region with the reflection of the dura-mater represented by the yellow line. The anterior clinoid process and optic pillar are identified in green (panels **A**–**I**), the optic nerve in gold, the anterior curve of the internal carotid artery (ICA) in red (panels **C**–**H**), the horizontal segment of the cavernous ICA (panel I) and the aneurysm itself by red arrows (panels **E**–**I**). Note that the posterior portion of the transitional aneurysm projects into the subarachnoid space — located above the distal dural ring (interrupted yellow line)

**Fig. 2:**
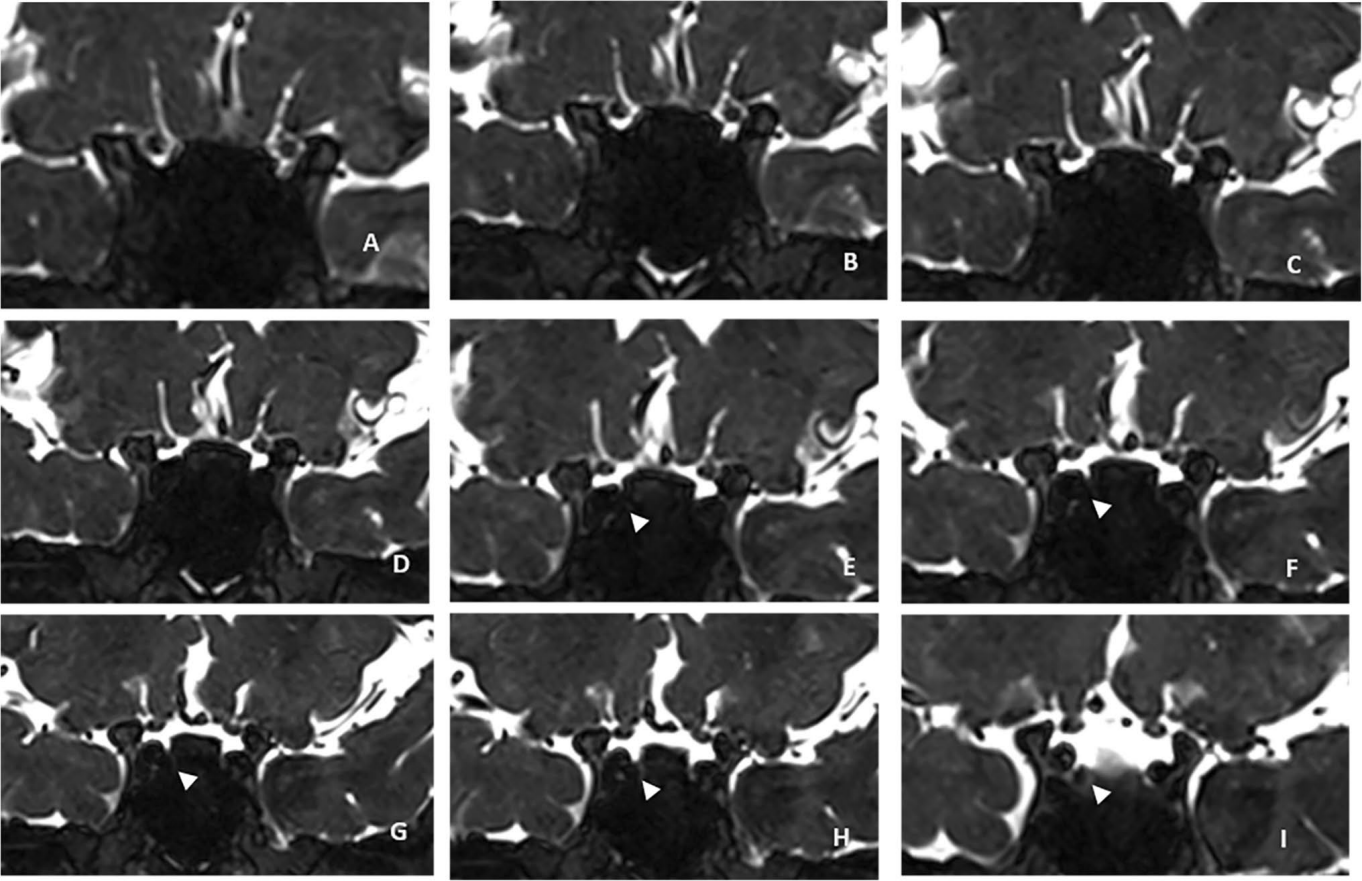
Coronal T2-weighted magnetic resonance imaging (MRI) scans acquired with a 3D fast spin-echo sequence showing anterior to posterior views of the anatomoradiological markers of the paraclinoid region as in Fig. 1 except that the markers are not highlighted (panels **A**–**D**). The transitional aneurysm is indicated by white arrow heads (panels **E**–**I**). Note that the posterior portion of the transitional aneurysm (black arrow) projects into the subarachnoid space

**Fig. 3:**
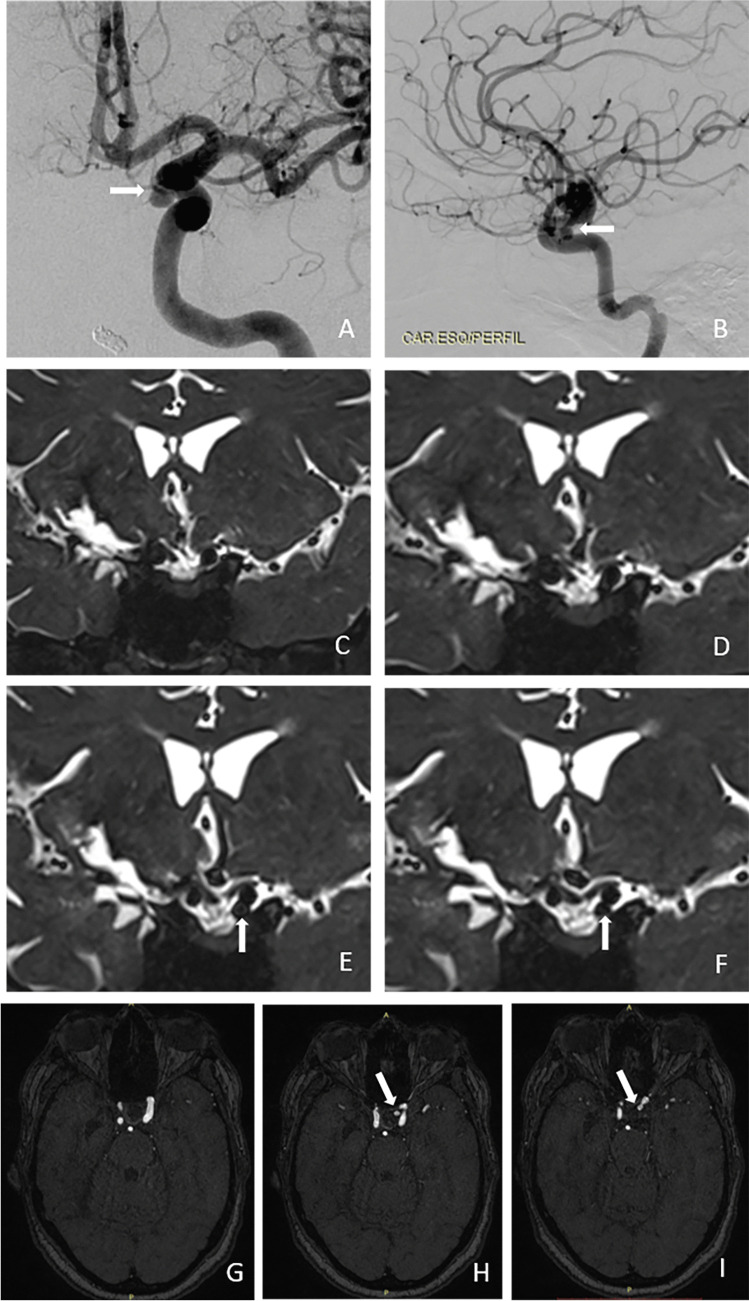
Cerebral digital subtraction angiography (DSA) of intradural aneurysm #22 (see Table 1) showing anteroposterior (panel **A**) and profile (panel **B**) views. Note the medial and inferior projection of the aneurysm (white arrows). Coronal T2-weighted magnetic resonance imaging (MRI) scans with a 3D fast spin-echo sequence showing anterior to posterior views of the anatomoradiological markers of the paraclinoid region and intradural aneurysm #22 (panels **C**–**F**). Note the hyper-intense signal from the cerebrospinal fluid (white arrows). In panel **G**–**I**, three-dimensional time-of-flight (TOF) magnetic resonance angiography was employed to enhance the view of the aneurysm (white arrows)

**Fig. 4:**
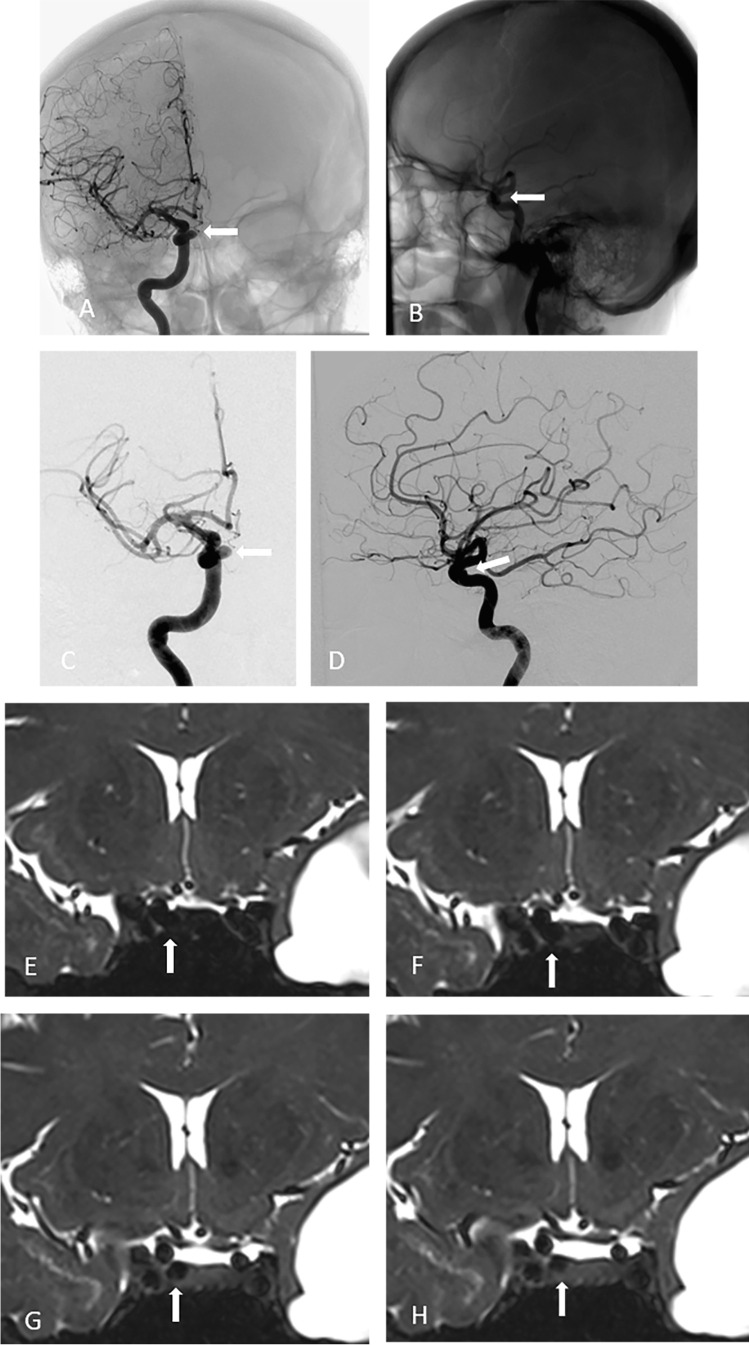
Cerebral digital subtraction angiography (DSA) of intracavernous aneurysm #19 (see Table 1) showing anteroposterior (panels **A** and **C**) and profile (panels **B** and **D**) views. Note the medial projection of the aneurysm (white arrows). Coronal T2-weighted magnetic resonance imaging (MRI) scans with a 3D fast spin-echo sequence showing anterior to posterior views of the anatomoradiological markers of the paraclinoid region and intracavernous aneurysm #19 (panels E–H). Note the hyper-intense signal from the cerebrospinal fluid around the distal dural ring and no contact between CSF and the aneurysm (arrow heads)

**Fig. 5:**
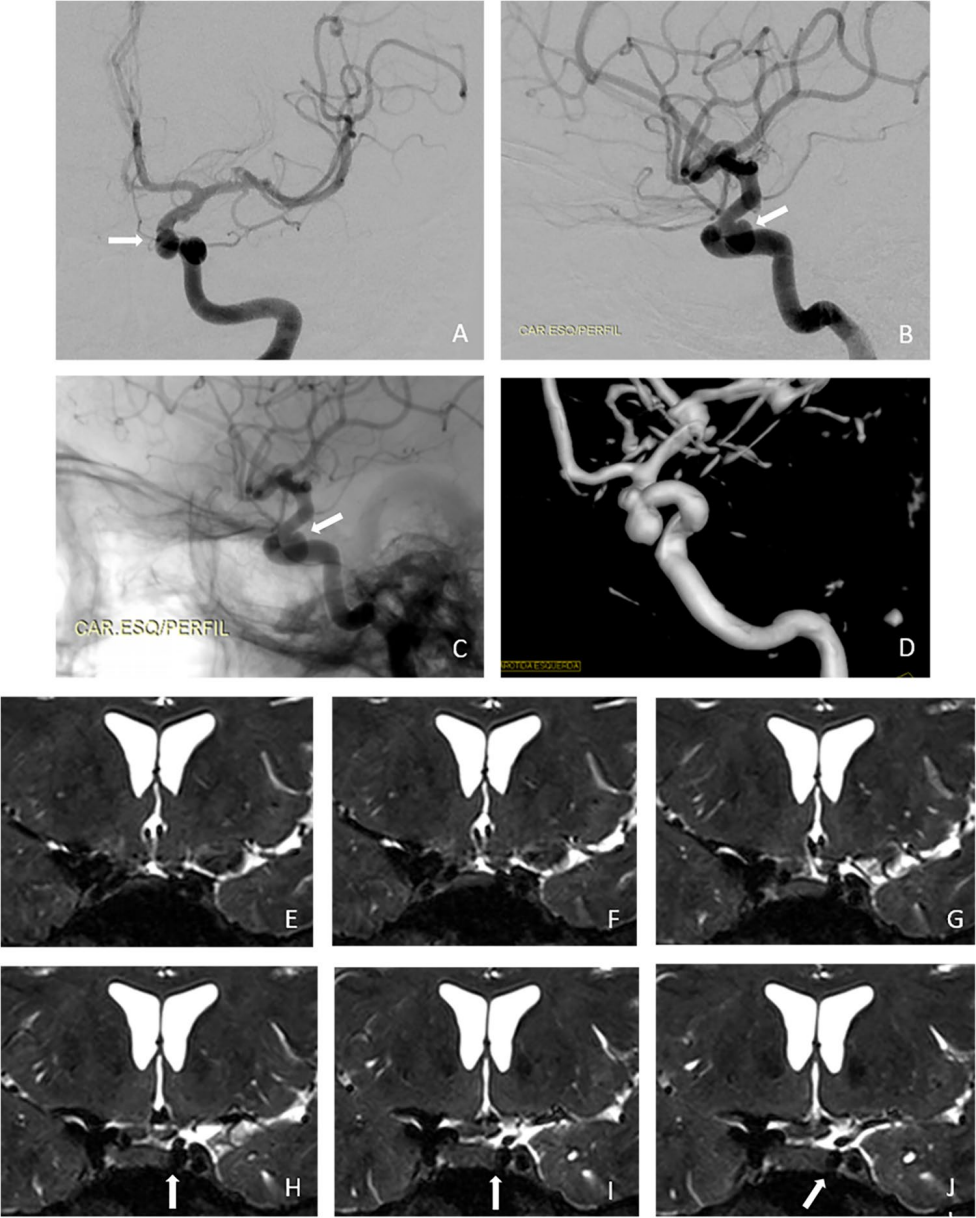
Cerebral digital subtraction angiography (DSA) of aneurysm #5 that caused intertechnique disagreement (see Table 1) showing anteroposterior (panel **A**) and profile (panel **B**) views. Note the medial projection of the aneurysm (white arrows). The relationship between the aneurysm and bony structures are shown (panels **C** and **D**). Coronal T2-weighted magnetic resonance imaging (MRI) scans with a 3D fast spin-echo sequence showing anterior to posterior views of the anatomoradiological markers of the paraclinoid region and the intracavernous (MRI)/transitional (M) aneurysm #5 (panels **G**–**J**; aneurysm — white arrows)

According to intraoperative findings, 40% (10/25) of the aneurysms were intracavernous, 8% (2/25) transitional and 52% (13/25) intradural, whereas MRI scans indicated 44.4% (11/25) of the aneurysms as intracavernous, 4% (1/25) as transitional and 52% (13/25) as intradural (Tables 1 and 3). Examples of intracavernous, transitional and intradural aneurysms are presented in Figs. 1, 2, 3, 4. Figure 5 presents a transitional aneurysm previously classified as intracavernous on MRI.

**Table 3:** Level of agreement between MRI and microsurgery regarding the classification of 25 paraclinoid aneurysms studied

Trichotomous classification of aneurysms						
		Microsurgery *n*(%)				MRI totals
		Intracavernous	Transitional		Intradural	
MRI	Intracavernous	9 (36)	1 (4)		1 (4)	11 (44.4)
	Transitional	1 (4)	0		0	1 (4)
	Intradural	0	1 (4)		12 (48)	13 (52)
Microsurgery totals		10 (40)	2 (8)		13 (52)	25 (100)
κ = 0.709; *P* < 0.001						
Dichotomous classification of aneurysms						
		Microsurgery *n*(%)				MRI totals
		Intracavernous		Non-cavernous^a^		
MRI	Intracavernous	9 (36)		2 (8)		11 (44.4)
	Non-cavernous	1 (4)		13 (52)		14 (56)
Microsurgery totals		10 (40)		15 (60)		25 (100)
κ = 0.754; *P* < 0.001						

^a^ Non-cavernous aneurysms comprised the intradural and transitional types

### Accuracy of MRI screening and intertechnique agreement

MRI sensitivity (probability of correctly indicating an aneurysm as non-cavernous) 86.7% (95%CI: 59.5–98.3). MRI specificity was 90.0% (95%CI: 55.5–99.8). PLR and NLR values were 8.7 (95%CI: 1.3–56.2) and 0.15 (95%CI: 0.04–0.6), respectively. The PPV and NPV values were 92.9% (95%CI: 66.1–99.8) and 81.8% (IC95%: 48.2–97.7), respectively.

For the subgroup of patients without previous SAH, sensitivity (92.3%; 95%CI: 63.9–99.8) and specificity (100%; 95%CI: 63–100) increased, while NLR decreased to 0.08 (95%CI: 0.01–0.51). PPV and NPV values reached 100% (IC95%: 73.5–100) and 88.9% (IC95%: 51.7–99.7), respectively.

The agreement was considered substantial for both dichotomous (κ = 0.754; *p* < 0.001) and trichotomous (κ = 0.709; *p* < 0.001) classifications (Table 3). For the subgroup of patients without previous SAH, agreement was even greater for the dichotomous classification (κ = 0.901; *p* < 0.001).

### Illustrative cases

The findings from MRI and microsurgery disagreed in aneurysms #5, #6, #13, and #20 (Table 1), all of which were ≤ 6 mm and medially orientated. Patients #5, #6, and #13 had previous SAH. Aneurysm #20 was the only one without previous SAH, which presented disagreement. Previously classified as intracavernous aneurysm on MRI, the DDR was opened before the location of the aneurysm had been ascertained. The practical translational meaning of our findings is: all patients with transitional or intradural aneurysms, eligible for surgical treatment, would be diagnosed correctly by MRI. Moreover, the calculated PPV of 100% means that all aneurysms classified as transitional or intradural (non-cavernous) by microsurgery were previously detected by MRI.

## Discussion

The MRI sensitivity was 86.7% while its specificity was 90%, thus demonstrating reliability for determining the location of paraclinoid aneurysms in relation to the DDR. MRI was efficient in ruling out the occurrence of non-cavernous aneurysms. Therefore, the probability of finding a non-cavernous aneurysm when a radiologist has classified an aneurysm as intracavernous based on MRI imaging is exceedingly small.

Aneurysms with previous SAH presented greater disagreement between the surgical findings and MRI impression. These divergences might be explained by possible arachnoiditis and consequent membranes thickening, or even some anatomical deformation of the paraclinoid region resulting from the SAH-induced inflammatory process. Interestingly, aneurysm #3 was also associated with a history of SAH from another aneurysm, but with concordant intracavernous location between MRI and surgery. For intracavernous aneurysms, we believe that the inflammatory process triggered by SAH might not influence MRI evaluation because they are not in contact with the subarachnoid space. Nevertheless, once SAH cases were excluded, MRI accuracy improved as well as its agreement with the intraoperative findings.

No studies have focused on the accuracy of diagnostic tests for determining the location of paraclinoid aneurysms in relation to the cavernous sinus. Reports available describe case series without sensitivity, specificity, and predictive values analysis [14, 16, 18, 20, 24–28]. For instance, Thines et al. [20] compared MRI with DSA and reported that MRI permitted the direct visualization of DDR and its relation to paraclinoid aneurysms. However, MRI accuracy compared to surgical findings or *post mortem* dissections was not reported.

The relationship of the aneurysm with the ophthalmic artery (OA) have been proposed as landmark for distinguishing between intra- and extradural paraclinoid aneurysms. However, the OA has extradural location in 2–8% of patients which is not reliable [29]. Other methods with CT angiography have been proposed for the paraclinoid aneurysms study based on bony limits of the DDR. Liao et al. [30] suggested the optic strut (OS) as a reliable landmark to determine an intradural growth of the aneurysm. The DDR is attached to the OS which might represent the anatomical limit between the intradural and extradural compartments. However, the OS represents only the anterior limit of the DDR that is not applied to all paraclinoid aneurysms that rise from different portions of the ICA. Therefore, Scerbak et al. [31] proposed a DDR plane based on four bony landmarks: (1) the intersection between the anterior clinoid and the ICA; (2) optic strut, (3) optico-carotid elevation, and (4) posterior clinoid process. They found 100% agreement between the radiological impression and surgical findings regarding the extension of the paraclinoid aneurysm in the subarachnoid space; however, these methods provide indirect evaluation of the DDR disposition. Our MRI method, on the other hand, provides direct coronal view of the DDR limits and the intradural portion of the aneurysm crossing the DDR limit.

The study described herein has some limitations. Our objective was to assess the accuracy, but not the reproducibility, of preoperative MRI. Since this is a single center study (one neuroradiologist and one neurosurgeon), further confirmation by multicentric studies is required. Although microsurgery was defined as the gold standard method against which MRI was tested, distraction during the surgical procedure could lead to aneurysm misclassification. Despite our status as a referral hospital for brain aneurysm treatment, it took 4 years for sufficient recruitment such that the learning curves for image interpretation and improvement of the microsurgery technique could be refined. The discrepancies observed between the two techniques appeared to be associated with anatomical deformities or MRI distortion caused by SAH-induced inflammation. While the exclusion of aneurysms with a history of SAH improved MRI accuracy, this could represent a potential limiting factor of the technique. Despite these shortcomings, the results of this study are robust and provide foundations for improvement of experimental design in future research.

MRI with 3D fast spin-echo sequence is a reliable and useful technique for determining the location of paraclinoid aneurysms in relation to the cavernous sinus and establishing the most appropriate therapeutic approach particularly for patients with paraclinoid aneurysm and no history of SAH.

## Data Availability

Data is fully available if necessary.
